# HIV-1-Specific Antibody Response and Function after DNA Prime and Recombinant Adenovirus 5 Boost HIV Vaccine in HIV-Infected Subjects

**DOI:** 10.1371/journal.pone.0160341

**Published:** 2016-08-08

**Authors:** Johannes S. Gach, Andrea Gorlani, Emmanuel Y. Dotsey, Juan C. Becerra, Chase T. M. Anderson, Baiba Berzins, Philip L. Felgner, Donald N. Forthal, Steven G. Deeks, Timothy J. Wilkin, Joseph P. Casazza, Richard A. Koup, Christine Katlama, Brigitte Autran, Robert L. Murphy, Chad J. Achenbach

**Affiliations:** 1 Department of Medicine, Division of Infectious Diseases, University of California Irvine, Irvine, California, United States of America; 2 Department of Medicine, Feinberg School of Medicine, Division of Infectious Diseases, Center for Global Health, Northwestern University, Chicago, Illinois, United States of America; 3 HIV/AIDS Division, San Francisco General Hospital and University of California San Francisco, San Francisco, California, United States of America; 4 Division of Infectious Diseases, Weill Cornell Medical College, New York, United States of America; 5 Vaccine Research Center, National Institute of Allergy and Infectious Diseases (NIAID), National Institutes of Health (NIH), Bethesda, Maryland, United States of America; 6 Department of Infectious Diseases, Pitié-Salpêtrière Hospital, Paris, France; 7 Department of Immunology, Pitié-Salpêtrière Hospital, Paris, France; Rush University, UNITED STATES

## Abstract

Little is known about the humoral immune response against DNA prime-recombinant adenovirus 5 (rAd5) boost HIV vaccine among HIV-infected patients on long-term suppressive antiretroviral therapy (ART). Previous studies emphasized cellular immune responses; however, current research suggests both cellular and humoral responses are likely required for a successful therapeutic vaccine. Thus, we aimed to understand antibody response and function induced by vaccination of ART-treated HIV-1-infected patients with immune recovery. All subjects participated in EraMune 02, an open-label randomized clinical trial of ART intensification followed by a six plasmid DNA prime (envA, envB, envC, gagB, polB, nefB) and rAd5 boost HIV vaccine with matching inserts. Antibody binding levels were determined with a recently developed microarray approach. We also analyzed neutralization efficiency and antibody-dependent cellular cytotoxicity (ADCC). We found that the DNA prime-rAd5 boost vaccine induced a significant cross-clade HIV-specific antibody response, which correlated with antibody neutralization efficiency. However, despite the increase in antibody binding levels, the vaccine did not significantly stimulate neutralization or ADCC responses. This finding was also reflected by a lack of change in total CD4+ cell associated HIV DNA in those who received the vaccine. Our results have important implications for further therapeutic vaccine design and administration, especially in HIV-1 infected patients, as boosting of preexisting antibody responses are unlikely to lead to clearance of latent proviruses in the HIV reservoir.

## Introduction

Antiretroviral therapy (ART) for HIV-infection improves health, prolongs life, and substantially reduces the risk of HIV transmission [1, 2]. Early ART is associated with a reduced latent viral reservoir and normalization of certain immune markers [3, 4]. Nevertheless, in the ART era, even when treated early, HIV remains a chronic progressive disease with persistent inflammation and immune activation leading to cardiovascular, hepatic, renal, and malignant diseases at higher rates than the general population [5]. Therefore, safe and effective preventive or therapeutic vaccines against HIV remain a global priority [6]. Effective HIV vaccines will likely need to induce both cellular and humoral HIV-specific immune responses. This has been studied through the delivery of multiple viral antigens including DNA plasmids and recombinant viruses [7–11]. Largely, vaccine clinical trials revealed strong CD8+ T cell responses and increases in HIV-specific antibodies without prevention of transmission or changes in HIV disease progression among those infected [6, 12]. Only one phase III clinical trial (RV144; clinicaltrials.gov: NCT00223080) conducted in Thailand has provided any evidence of protection with an estimated efficacy of 31.2% against the acquisition of HIV [13, 14]. While the ALVAC-HIV and AIDSVAX B/E (gp120) vaccine products in the Thai trial did not induce broadly neutralizing antibodies or robust cytotoxic T-lymphocyte responses it stimulated robust HIV-specific antibody-dependent cellular cytotoxicity (ADCC) responses among those protected from infection [15–17]. Post-hoc analyses showed that non-neutralizing antibodies to the C1 and V1/V2 regions of envelope correlated inversely with the risk of HIV infection and that high levels of ADCC IgG were associated with a reduced risk of HIV acquisition in the presence of low HIV-specific IgA antibody levels. [18]. ADCC has also been postulated as a mechanism by which infusion of broadly neutralizing HIV-specific monoclonal antibodies (e.g. VRC01) could eliminate latently infected cells in ART-treated patients [15, 19].

We have limited information on antibody response and function after administration of HIV vaccines to individuals on effective long-term ART. A recent phase I/II clinical trial of ART-treated individuals vaccinated with an HIV DNA vaccine (VRC-HIVDNA 009-00-VP, AIDS Clinical Trials Group (ACTG) 5187 study) showed poor immunogenicity with low CD4+ and CD8+ IFN-γ ELISpot responses; HIV-specific antibody responses, including ADCC, were not reported [7]. In another trial, VRC101, ART-suppressed adults administered HIV DNA prime-rAd5 boost vaccine (containing VRC-HIVDNA016-00-VP and VRC-HIVADV014-00-VP) had no changes in pooled clade A, B, or C envelope antibody titers one month after vaccination, except for a non-significant increase in binding to peptides in the V3 loop [20]. They did not report on whether the vaccine altered antibody neutralization or ADCC. Therefore, in this study, we performed a more comprehensive evaluation of HIV-specific antibody titer, neutralization, and ADCC after administration of an HIV DNA prime and rAd5 boost vaccine to ART-treated patients in a phase II, randomized, clinical trial (EraMune 02; clinicaltrials.gov: NCT00976404) [21]. We aimed to improve our understanding of HIV-specific antibodies in ART-treated patients and whether vaccine products designed to elicit cellular immunity enhance antibody response or function.

## Materials and Methods

### Study design and population

We performed this substudy on all subjects enrolled in the EraMune 02 multicenter, open-label, randomized phase II clinical trial (clinicaltrials.gov NCT00976404) of ART intensification alone or with DNA prime-rAd5 boost vaccination [21]. EraMune 02 included chronically HIV-1 infected participants aged 18 to 70 years who were on suppressive ART (plasma HIV RNA <500 copies per ml for at least three years and below the limit of detection within the past year) with a current CD4+ T cell count ≥350 per μL. Participants had cell-associated HIV DNA between 10 and 1,000 copies per 10^6^ PBMCs and we excluded subjects who had a serum Ad5 90% neutralization antibody titer >250. The study protocol was approved by the Investigational Review Board of Northwestern University and by IRBs at the two other participating study sites (University of California San Francisco and Cornell University). We obtained written informed consent from all subjects including banking of plasma and PBMCs for future investigations. For this antibody substudy we analyzed banked plasma from baseline, week 36, and week 56 in both study arms.

### Intervention and vaccination schedule

After enrollment, all subjects remained on their baseline ART and received raltegravir 400 mg twice-daily (provided by Merck & Co. US) and maraviroc 150, 300 or 600 mg twice daily (provided by Pfizer Inc. and ViiV Healthcare) for 56 weeks. Subjects randomized to the RAL/MVC/vaccine arm also received 4 mg VRC-HIVDNA016-00-VP priming (DNA prime, consisting of envA, envB, envC, gagB, polB, nefB) vaccinations at week 8, 12 and 16, followed by 10^10^ particle units of VRC-HIVADV014-00-VP (rAd5 boost, consisting of envA, envB, envC, gagB, polB, nefB) (provided by National Institute of Allergy and Infectious Diseases (NIAID) Vaccine Research Center (VRC)) as a boost vaccination at week 32. Both vaccines have been described previously [22]. All vaccinations were given intramuscularly in a 1 mL volume. The Biojector 2000 injection system was used to administer VRC-HIVDNA016-00-VP; a needle and syringe were used to administer VRC-HIVADV014-00-VP.

### Microarray analysis

Binding of patient plasma samples were evaluated on a protein microarray platform [23] containing three multi-clade gp140 HIV-1 specific proteins: UG37 (clade A), SF162 (clade B), and CN54 (clade C) [24–27]. Proteins were printed at a concentration of 0.01 mg/mL onto nitrocellulose coated glass FAST slides (Whatman). Prior to array probing, plasma was diluted 1/100 in Protein Array Blocking Buffer (Whatman, GE Healthcare) containing *E*. *coli* lysate at a final concentration of 10 μg/mL and incubated at room temperature (RT) for 1 h. Arrays were rehydrated and blocked in blocking buffer for 30 min and then probed with the pretreated sera overnight (o/n) at 4°C. After washing several times, slides were incubated with a biotin-SP conjugated goat anti-human IgG Fc specific secondary antibody (Jackson ImmunoResearch) diluted 1/200 in blocking buffer. Bound antibodies were detected by 1 h incubation with streptavidin-conjugated SureLight P-3 tertiary reagent (Columbia Biosciences) diluted 1/200 in blocking buffer. Arrays were examined with a Perkin Elmer ScanArray Express HT confocal laser scanner at a wavelength of 670 nm and signal intensities were quantified using ProScanArray Express software (Perkin Elmer). All signal intensities were corrected for spot-specific background.

### Antibody purification and neutralization assay

IgG from patient plasma samples was purified as described previously [28] and tested for neutralization activity using pseudotyped HIV-1_JR-FL_ and HIV-1_SF162_ virions. VSV-g pseudotyped HIV-1 virions were used as a control for antibody independent neutralization activity of the purified IgG fractions. Virus production and neutralization assays were performed according to Gach and colleagues [29]. In brief, 5 x 10^5^ human embryonic kidney 293T cells (ATCC, CRL-3216) were co-transfected with 4 μg of the HIV-1 Env-deleted backbone plasmid pSG3ΔEnv (NIHARRRP; contributed by J. Kappes and X. Wu) and 2 μg of the respective HIV Env or VSV-g complementation plasmid using polyethyleneimine (PEI) as a transfection reagent (DNA/PEI ratio of 1/3). After 48 h post transfection cell culture supernatants were harvested und used for subsequent studies. For virus neutralization assays pseudotyped virus was added at a 1:1 ratio to serially diluted purified IgG samples and incubated at 37°C. After 1 h TZM-bl reporter cells (ATCC, PTA-5659) were added (1:1 by volume) at 1×10^4^ cells/well in a final concentration of 10 μg/mL DEAE-dextran and incubated for 48 h at 37°C. The cells were then washed, lysed, and finally developed with luciferase assay reagent according to the manufacturer’s instructions (Promega). Luminescence in relative light units (RLU) was measured using a Synergy 2 microplate luminometer (BioTek). All experiments were performed at least in duplicate. The extent of virus neutralization in the presence of antibody was determined at the 50% inhibitory concentration (IC_50_) in the absence of Ab [30].

### ADCC assay

Purified antibody fractions were further analyzed for ADCC functions. PBMCs were isolated from human whole blood obtained from healthy donors using a Ficoll-Paque Premium (GE Healthcare) gradient and washed twice with PBS. In the meantime HIV-1_JR-FL_ and HIV-1_SF162_ infected CEM NKr-CCR5 cells or trimeric BG505 SOSIP.664 gp140 envelope coated (20 μg/mL) CEM NKr-CCR5 cells were opsonized for 1 h at 4°C with 50 μg/mL of purified patient antibody. Isolated PBMCs were then added to antibody opsonized CEM NKr cells at an effector-to-target ratio of 50:1 and incubated for 4 h at 37°C. After 4 h the cell-antibody-mixture was transferred into 96-white well plates and release of a distinct intracellular protease activity (dead cell protease) was measured with a CytoTox-Glo cytotoxicity assay kit according to the manufacturer’s instructions (Promega). Percent cytotoxicity was calculated as described elsewhere [31, 32]: Percent cytotoxicity = (experimental—effector spontaneous—target spontaneous) / (target maximum—target spontaneous) x 100. “Experimental” corresponds to the signal measured in a treated sample, “effector spontaneous” corresponds to the signal measured in the presence of PBMCs alone, “target spontaneous” corresponds to the signal measured in the presence of opsonized CEM NKr-CCR5 cells alone, and “target maximum” corresponds to the signal measured in the presence of lysed CEM NKr-CCR5 cells. Experiments were performed at least twice in duplicate.

### Enzyme linked immunosorbent assay (ELISA)

Ninety-six well plates were coated with 100 ng/well of HIV-1gp140_UG37_ (clade A), HIV-1gp140_SF162_ (clade B), and HIV-1gp140_CN54_ (clade C) and incubated o/n at 4°C. Plates were blocked (4% non-fat dry milk in PBS containing 0.05% Tween20), washed, and probed with serially diluted purified IgG fractions from baseline and week 56 starting at a concentration of 20 μg/mL. After 1 h plates were washed and incubated with a goat anti human Fc specific HRP labeled secondary antibody. Bound antibodies were detected and read as described elsewhere [33]. ELISA experiments were performed in duplicate.

### Statistical analysis

Baseline subject characteristics were compared between vaccine and non-vaccine arms using unpaired Wilcoxon rank-sum and Fischer exact tests for continuous and categorical variables, respectively. To assess significant differences between the different time points (i.e. baseline, week 36 and week 56) within each group (i.e. vaccine and non-vaccine), microarray data, neutralization data, and ADCC data were log_10_ transformed and analyzed by paired comparisons using repeated measures ANOVA (RM ANOVA) followed by post hoc comparisons (Dunnett’s multiple comparison test). To prevent false positive results, the RM ANOVA models were further tested for the sphericity assumption in order to determine if the type 1 error rate is larger than the nominal level. For the comparison of microarray data with neutralization data or ADCC data as well as ELISA data with neutralization data or ADCC data, we used a nonparametric Spearman correlation to analyze potential correlations between two continuous variables. Since not all of the data showed a clear linear correlation but rather a demarcation of the scatter plot we bifurcated the data sets into < median and > median as we wanted to understand the impact of high and low antibody signals/titers for antibody mediated functions. Mann-Whitney test was used to analyze significant differences between quartiles. Spearman correlation was further used to analyze correlations between neutralization (IC_50_) values and ADCC activity. All descriptive, comparative and correlative statistics were performed using Graph Pad Prism 7.0. Test results were considered statistically significant for two-sided p-value <0.05.

## Results

### Microarray analysis revealed different antibody binding patterns to HIV-1gp140 envelopes after vaccination

Between July 2010 and October 2011, we enrolled 28 subjects on suppressive ART (14 in each arm) with no detectable antibodies against Ad5. All of the study participants were men, 20/28 (71%) were white, and the median age was 50 years (IQR 46, 55) (Table 1). Subjects had been on any ART for a median of 13 years (IQR 8, 19) with median time of undetectable HIV RNA levels (<50 copies per mL) of 2.6 years (IQR 2.2, 3.0). The median nadir CD4+ cell count was 202 cells per μL (IQR 88, 280), median baseline CD4+ cell count was 636 cells per μL (IQR 485, 790) and median baseline total cell-associated HIV DNA was 170 copies per 10^6^ PBMCs (IQR 60, 361).

**Table 1:** Characteristics of subjects enrolled in EraMune 02 and analyzed in antibody substudy. Data are number (%) or median (IQR). ART = antiretroviral therapy. NRTI = nucleoside reverse transcriptase inhibitor. NNRTI = non-nucleoside reverse transcriptase inhibitor. PI/r = protease inhibitor boosted with low-dose ritonavir. PI = protease inhibitor. The p-values were calculated between vaccine and non-vaccine arm.

	Overall (n = 28)	Non-Vaccine (n = 14)	Vaccine (n = 14)	p-value*
**Male sex**	28 (100%)	14 (100%)	14 (100%)	
**White race**	20 (71%)	11 (79%)	9 (64%)	0.678
**Age (years)**	50 (46, 55)	49 (46, 55)	50 (46, 55)	0.910
**Time on ART (years)**	13 (8, 19)	13 (8, 19)	13 (6, 19)	0.960
**Prior AIDS events**	6 (21%)	3 (21%)	3 (21%)	1.000
**Nadir CD4 cell count (cells per μL)**	202 (88, 280)	220 (146, 419)	179 (50, 219)	0.057
**Baseline CD4+ T cell count (cells per μL)**	636 (485, 791)	686 (501, 880)	563 (468, 718)	0.246
**Baseline CD8+ T cell count (cells per μL)**	672 (516, 817)	625 (475, 925)	719 (535, 796)	0.571
**Ad5 90% neutralization titer**	12 (12, 41)	18 (12, 68)	12 (12, 38)	0.306
**Duration with HIV RNA < 50 copies per mL (years)**	2·6 (2·2, 3·0)	2·4 (2·3, 3·1)	2·7 (2·1, 3·0)	0.960
**Baseline HIV DNA (copies per 10**^**6**^ **PBMCs)**	170 (60, 361)	97 (47, 352)	228 (98, 383)	0.482
**ART regimen**				0.214
•2 NRTIs + PI/r	11 (39%)	4 (39%)	7 (50%)	
• 2 NRTIs + NNRTI	15 (54%)	10 (71%)	5 (36%)	
• NRTI + NNRTI + PI/r	1 (4%)	0 (0%)	1 (7%)	
• NNRTI + PI	1 (4%)	0 (0%)	1 (7%)	

* Comparison of vaccine and non-vaccine arms

**Fig 1:**
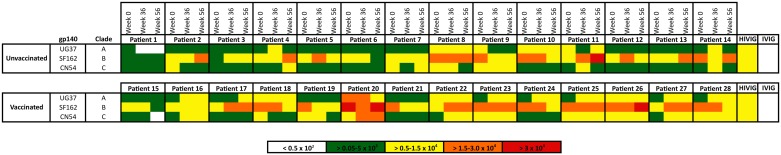
Microarray analysis of HIV-1 infected patient samples. The microarray chip was probed with unvaccinated (non-vaccine arm) and vaccinated (vaccine arm) patient plasma samples (week 0, week 36, and week 56) at a dilution factor of 1:100. HIV-1 specific gp140 envelope proteins were printed at a concentration of 0.01 mg/mL, which corresponds to 0.01 ng per spot. HIVIG and IVIG were included as a positive control and negative control, respectively. Antibody signal intensities were color-coded using green (>0.05–0.5 x 10^3^) for weak, yellow (>0.5–1.5 x 10^4^) for intermediate, orange (>1.5–3.0 x 10^4^) and red (>3.0 x 10^4^) for strong interaction. Non-specific binding was indicated as white (<0.5 x 10^2^) boxes. Raw data is indicated in S3 Fig.

Plasma samples from both study arms were analyzed for specific binding against three HIV-1 specific envelope antigens, including clade A gp140_UG37_ (EnvA), clade B gp140_SF162_ (EnvB), and clade C gp140_CN54_ (EnvC). Based on initial microarray experiments, the HIV-1 specific envelope proteins were printed at a concentration of 0.01 mg/mL or 0.01 ng per spot. The heat map of our microarray analysis, shown in Fig 1, revealed a unique antibody binding profile for each tested subject. First, we evaluated the median antibody signals of all printed envelope proteins together (i.e. cross-clade), and then analyzed each sub-clade individually.

### Clade A, and clade C-specific antibody levels were significantly elevated after vaccination

**Fig 2:**
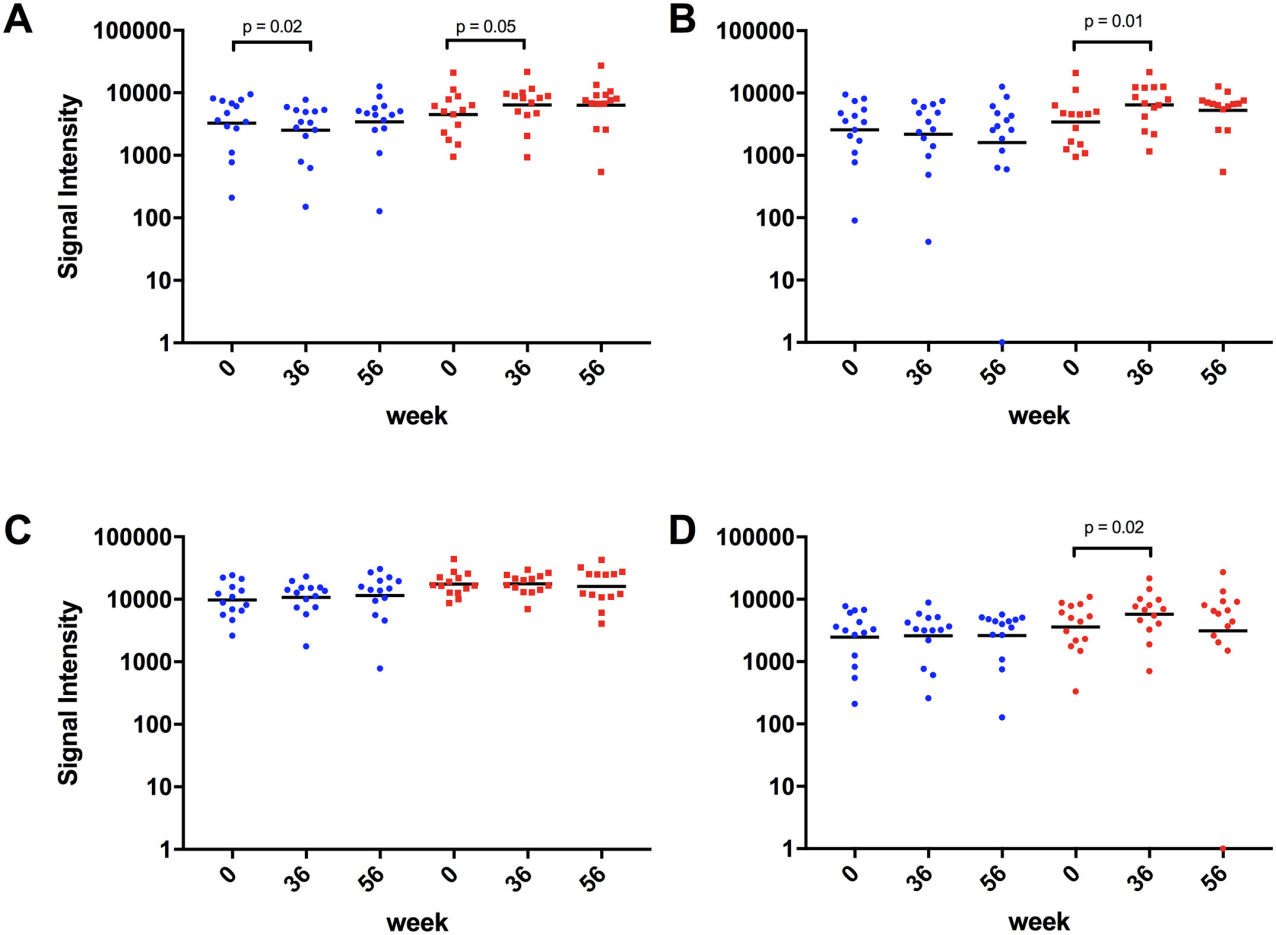
Evaluation of antibody binding signal intensities. Microarray signal intensities were analyzed for differences within the non-vaccine arm (blue scatter plots) as well as the vaccine arm (red scatter plots). Signals deriving from the cross clade response (A), the clade A gp140 response (B), the clade B gp140 response (C), and clade C gp140 response (D) were evaluated and analyzed for significant differences using a RM ANOVA and Dunnett’s multiple comparisons test (GraphPad Prism 7.0). The cross clade (A) signal intensities at week 36 (p = 0.05) in the vaccine arm and the clade A (B) and clade C (D) signal intensities at week 36 (p = 0.01) and p = 0.02, respectively) in the vaccine arm were significantly higher compared to baseline. No significant differences were found in the clade B (C) signal intensities in the vaccine arm.

As indicated in Fig 2A, we found significantly different (p = 0.0429, RM ANOVA) cross clade HIV-1 specific antibody binding signals between the three time points (week 0, week 36, and week 56) in subjects that received the HIV-rAd5 boost regimen. Dunnett’s multiple comparisons test revealed a significant increase in signal intensity at week 36 (42% signal increase; p = 0.0466) and a non-significant increase at week 56 (41% signal increase; p = 0.1169) compared to baseline. Antibody-binding signals in the non-vaccine arm revealed also a significant difference between the time points (p = 0.0223; RM ANOVA). However, contrary to the vaccine group, signals dropped by 23% at week 36 and remained relatively constant at week 56 (5% signal increase) compared to baseline signals (Fig 2A).

Next, we evaluated antibody-binding signals of both arms against the individual antigens of each clade. We found a significant difference in signal intensities against EnvA in the vaccinated arm (p = 0.0088; RM ANOVA) especially at week 36 (89% signal increase; p = 0.0054; Dunnett’s multiple comparisons test) and a non-significant increase at week 56 (54% signal increase; p = 0.1079; Dunnett’s multiple comparisons test), compared to baseline signals (Fig 2B). On the contrary, as indicated in Fig 2B, signal intensities of the non-vaccine arm declined over time (i.e. 15% signal decrease at week 36 and 37% decrease at week 56).

No significant differences in signal intensity between baseline and post vaccination time points were found against EnvB within the vaccinated group (Fig 2C). We noticed only subtle changes in antibody signal levels at week 36 (1% signal increase) and 56 (9% signal decrease). However, antibody signals at week 36 (9% signal increase) and week 56 (17% signal increase) were elevated in the non-vaccine arm. EnvB antibody signal intensities measured at baseline in the vaccine arm were almost 2-fold higher than signal intensities measured in the non-vaccine arm. When we evaluated EnvC-specific antibody signal intensities, we observed a significant (p = 0.0242; Dunnett’s multiple comparisons test) increase in signal intensities at week 36 (59% signal increase) and a non-significant decrease at week 56 (14% signal increase, p = 0.9306; Dunnett’s multiple comparisons test), versus baseline signal levels (Fig 2D). Additionally, as indicated in Fig 2D, signals in the non-vaccine arm revealed nearly consistent intensities at week 36 and week 56 (5% signal increase and 5% signal increase, respectively).

### Vaccination did not significantly enhance neutralization potency against clade B virions

**Fig 3:**
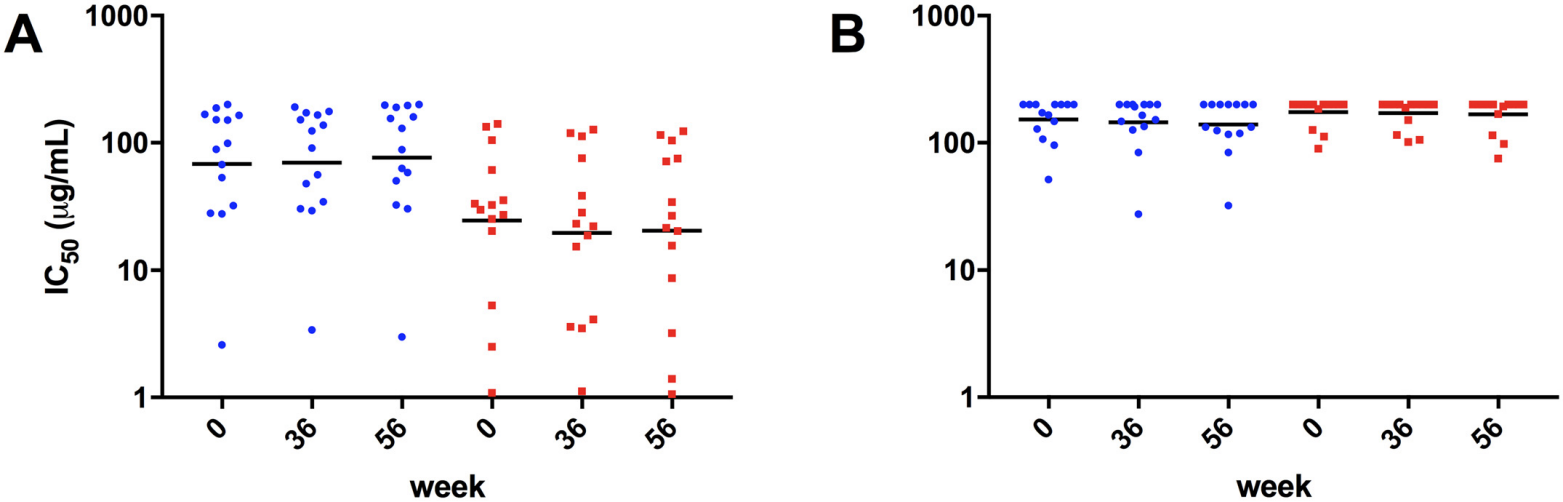
Neutralization efficiency of purified IgG fractions from study participants. The extent of virus neutralization in the presence of antibody was determined at the 50% inhibitory concentration (IC_50_) in the absence of antibody. Purified antibodies were tested at an initial concentration of 200 μg/mL against clade B viruses HIV-1_SF162_ (A) and HIV-1_JR-FL_ (B). All neutralization experiments were performed in duplicates. The negative control HIV-1/VSV-g revealed no neutralization activity at the highest concentration tested.

Neutralizing activity of purified IgG fractions were investigated from all study participants against two clade B isolates HIV-1_SF162_ and HIV-1_JR-FL_ as well as a negative control HIV-1 VSV-g. As expected, no neutralization activity against HIV-1 VSV-g-pseudotyped virions was observed after IgG purification (data not shown). IC_50_ values for both study groups and viruses are depicted in Fig 3. We found slightly lower IC_50_ values at week 36 (19.5 μg/mL) and week 56 (20.3 μg/mL), compared to baseline (24.4 μg/mL) against the sensitive Tier 1 isolate HIV-1_SF162_ in the vaccine arm (Fig 3A). None of the observed differences were significant according to RM ANOVA and Dunnett’s multiple comparisons test. In contrast, IC_50_ values of the non-vaccine arm (Fig 3B) slightly increased over time from 68.4 μg/mL at baseline to 76.4 μg/mL at week 56. When we analyzed IC_50_ values of the Tier 2 isolate HIV-1_JR-FL_ we found only subtle differences between the three time points in both the vaccine and non-vaccine arm. IC_50_ values were ranging from 174.2 μg/mL at baseline to 171.0 μg/mL at week 36, and 167.9 μg/mL at week 56. Similarly, neutralization potency slightly improved against HIV-1_JR-FL,_ over time in the non-vaccine arm with IC_50_ values dropping from 152.4 μg/mL at baseline to 139.3 μg/mL at week 56 (9% improved neutralization capacity compared to baseline).

Next, we analyzed changes in Tier 2 neutralization over time in a subset of subjects with HIV-1_JR-FL_ IC_50_ values below 200 μg/mL (n = 6 vaccine arm; n = 8 non-vaccine arm). As indicated in S1 Fig we found increased potency against HIV-1_JR-FL_ in the vaccine arm at week 36 (138.4 μg/mL) and week 56 (132.8 μg/mL) compared to baseline (145.2 μg/mL). None of the observed differences were significant according to our RM ANOVA and Dunnett’s multiple comparisons test analysis. The non-vaccine arm exhibited elevated neutralization potency at week 56 (106.4 μg/mL) compared to baseline (124.5 μg/mL) and week 36 (113.5 μg/mL).

### No significant increase in ADCC was observed in the vaccinated group

**Fig 4:**
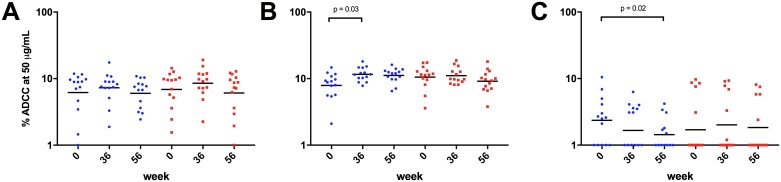
ADCC activity of study samples. ADCC activity was measured using purified IgG fractions against HIV-1_SF162_ (A) and HIV-1_JR-FL_ (B) infected cells. Additionally, antibodies were evaluated against a clade A BG505 SOSIP.664 gp140 trimer coated cells (C). RM ANOVA and Dunnett’s multiple comparisons test revealed significantly enhanced ADCC activity only in the non-vaccine arm at weeks 36 (p = 0.03), compared to baseline (B). A significant decrease (p = 0.02) in ADCC activity was observed with the BG505 SOSIP.664 gp140 trimer coated cells in the non-vaccine group (C).

We further studied antibody effector functions by performing ADCC assays with HIV-1_SF162_ and HIV-1_JR-FL_ infected CEM.NKr-CCR5 cells as well as BG505 SOSIP.664 gp140 decorated CEM.NKr-CCR5 cells. As indicated in Fig 4A, % ADCC of HIV-1_SF162_ infected cells was following a similar trend in both study arms with increasing activity at week 36 (24% increase in the vaccine arm versus 18% increase in the non-vaccine arm) and declining activity at week 56 (12% decrease in the vaccine arm versus 3% decrease in the non-vaccine arm) compared to baseline. A different trend was observed with HIV-1_JR-FL_ infected cells where the non-vaccine arm revealed significantly different (p = 0.0178; RM ANOVA) ADCC activity between the analyzed time points. Dunnett’s multiple comparisons test exhibited a significant increase in ADCC activity at week 36 (47% increase; p = 0.0297) and a non-significant increase at week 56 (41% increase, p = 0.0610), compared to baseline (Fig 4B). In contrast, as indicated in Fig 4B, we found similar ADCC activities in the vaccine arm at week 36 (5% ADCC) and a slightly reduced ADCC activity at week 56 (13% ADCC), compared to baseline activity (11.3% ADCC). ADCC activity against the clade A BG505 SOSIP.664 gp140 coated CEM cells significantly declined (39%) at week 56 (p = 0.0220; Dunnett’s multiple comparisons test) in the non-vaccine arm (Fig 4C), whereas we observed a slight increase (18%) in ADCC activity at week 36 in the vaccine arm. This trend, albeit more pronounced, was also observed after excluding patients with no ADCC activity against the clade A SOSIP trimer (control group (n = 9) and vaccine group (n = 7)) (S2 Fig).

### Presence of high HIV-1SF162-specific antibody titers indicates neutralization potency and ADCC activity

**Fig 5:**
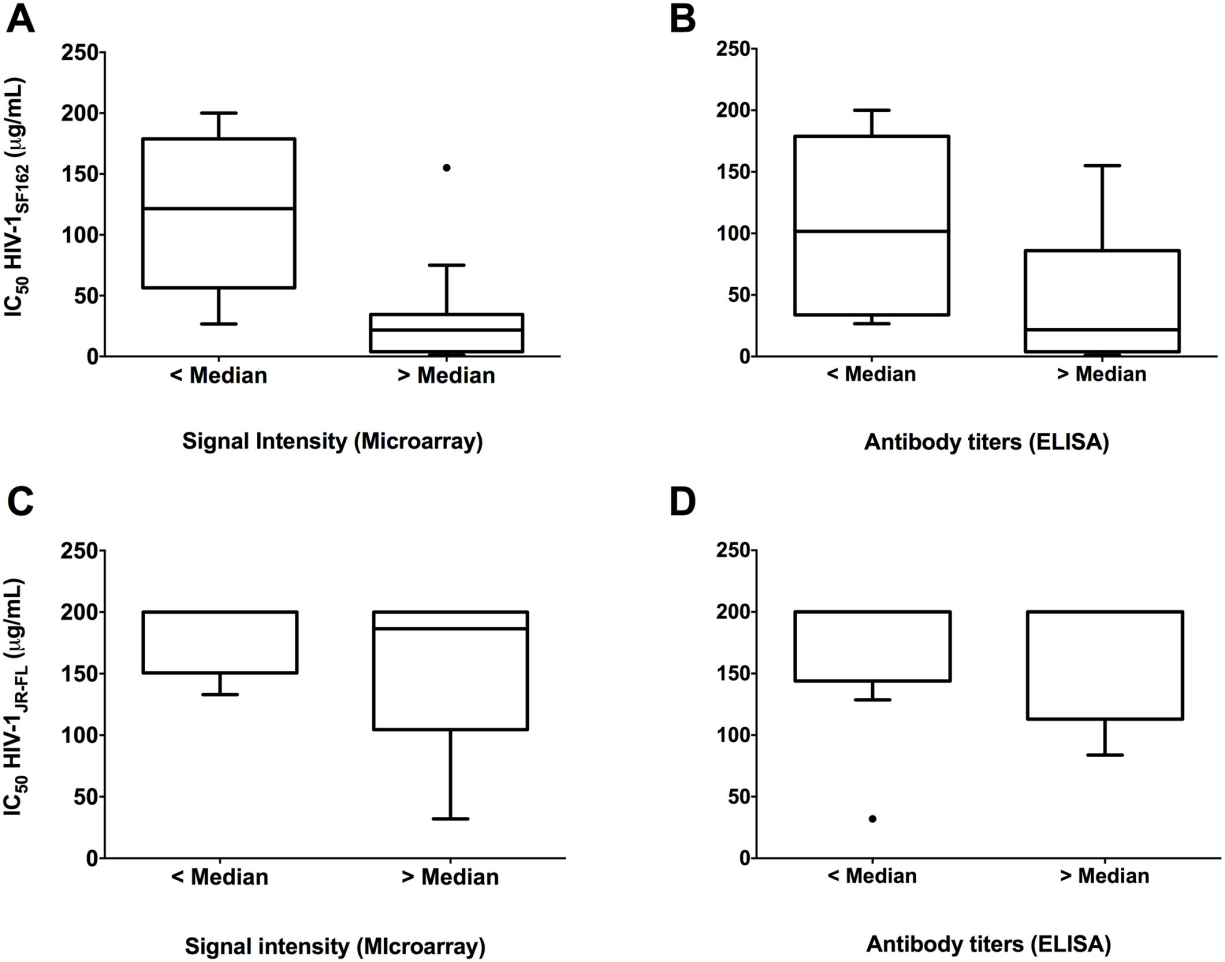
Correlation between virus neutralization and antibody signal intensities as well as ELISA antibody titers. Median cross clade signal intensities and ELISA antibody titers were bifurcated into two specific groups (< median and > median) and compared to neutralization titers. Mann-Whitney test exhibited significantly lower HIV-1_SF162_ IC50’s in subjects with higher microarray antibody signals (p = 0.0003) (A) or higher ELISA titers (p = 0.0094) (B). No differences were observed between HIV-1_JR-FL_ and antibody signals (C) or ELISA titers (D).

In order to evaluate potential relationships between neutralization efficiency (HIV-1_SF162_ and HIV-1_JR-FL_) and cross clade signal intensities of both arms together at all time points we bifurcated the median cross clade signal intensities into two specific groups: < median and > median. As shown in Fig 5A, we found significantly lower HIV-1_SF162_ IC_50_ values in samples with higher signal intensity (p = 0.0003; Mann-Whitney test). A similar relationship (p = 0.0094; Mann-Whitney test) was observed when we compared HIV-1_SF162_ neutralization with the cross clade ELISA titers (Fig 5B). Similar relationships between neutralization of HIV-1_JR-FL_ and signal intensity or antibody titer were not apparent. Fig 5C and 5D.

**Fig 6:**
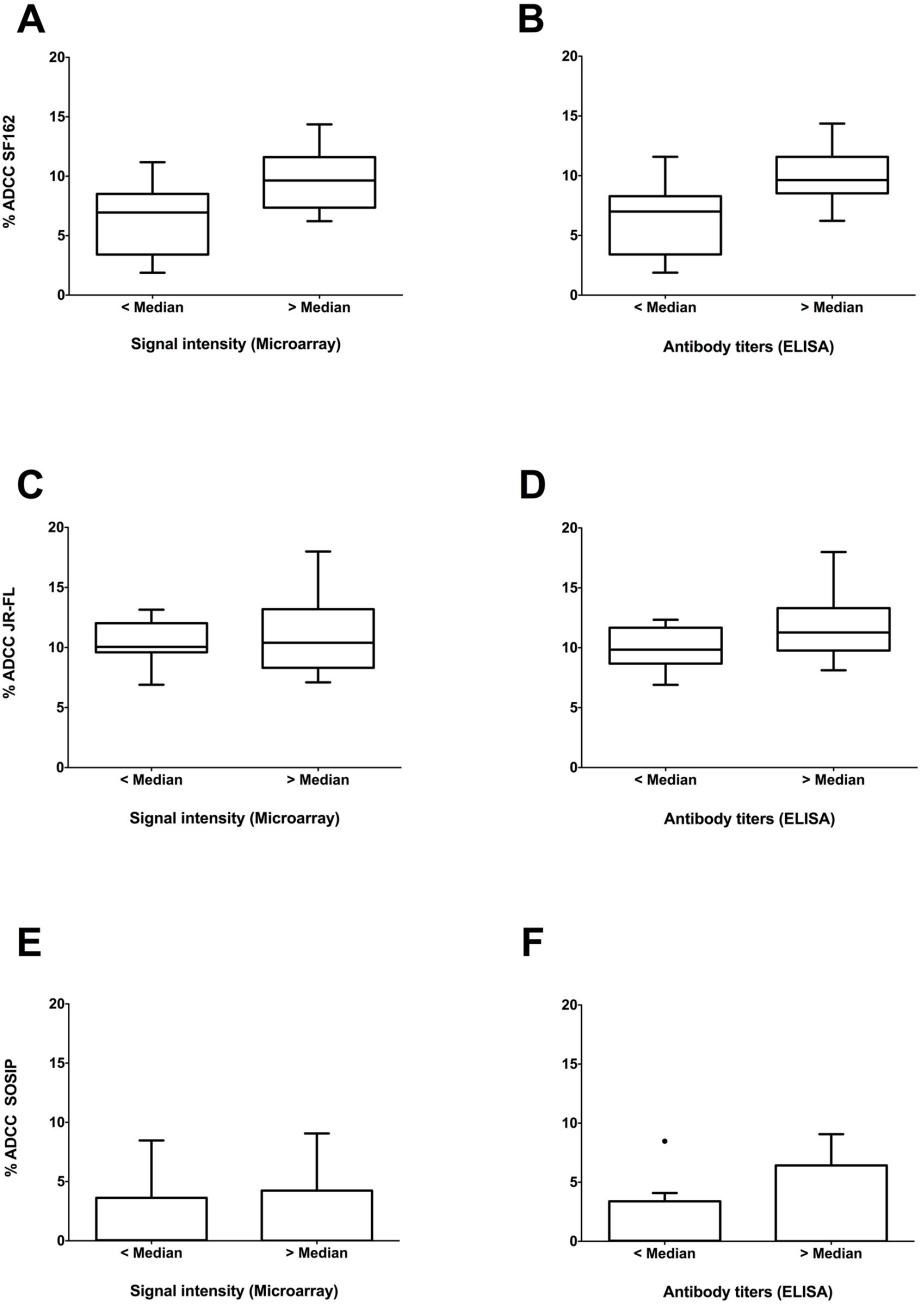
Correlations between ADCC activity and antibody signal intensities as well as ELISA antibody titers. Median cross clade signal intensities and ELISA antibody titers were stratified into two specific groups (< median and > median) and compared to %ADCC activity. Significant correlations were found between patient samples with high ADCC activity against HIV-1_SF162_ and high median cross clade signal intensities (p = 0.004) (A) as well as high median cross clade ELISA titers (p = 0.004) (B). A clear trend towards higher ADCC activity against HIV-1_JR-FL_ infected cells was observed with higher antibody signals (C) or ELISA titers (D). No correlation was found between ADCC of SOSIP decorated cells and median cross clade signals (E) and ELISA titers (F).

We next measured correlations between ADCC activity and cross clade signal intensities or ELISA titers. As indicated in Fig 6A we found significantly higher ADCC activity against HIV-1_SF162_ infected cells in samples with higher signal intensity (p = 0.004; Mann-Whitney test). A similar correlation (p = 0.004; Mann-Whitney test) was found between ADCC activity against HIV-1_SF162_ infected cells and cross clade ELISA titers (Fig 6B). In contrast, no correlations were observed between ADCC activity against HIV-1_JR-FL_ infected cells and antibody signals or ELISA titers (Fig 6C and 6D). As indicated in Fig 6E and 6F, no significant correlations were found between ADCC activity against SOSIP trimer decorated cells and antibody signals or ELISA titers.

### Neutralization activity correlates with ADCC activity

In a final analysis, we evaluated direct correlations between IC_50_’s and ADCC activity. We found significant negative correlations between %ADCC_SF162_ and IC_50_ HIV-1_JR-FL_ (p = 0.0006; r = -0.366; Spearman) as well as %ADCC_SF162_ and IC_50_ HIV-1_SF162_ (p<0.0001; r = -0.529; Spearman). In contrast, no correlations were found between %ADCC_SOSIP trimer_ and IC_50_’s or %ADCC_JR-FL_ and IC_50_’s. However, in the case of %ADCC_JR-FL_ we observed a trend towards higher ADCC activity with higher neutralization potency against HIV-1_JR-FL_ and HIV-1_SF162_.

## Discussion

Our rationale to study longitudinal humoral immune responses elicited by this DNA prime/rAd5 boost vaccine combination was to improve our understanding of antibody response and function in the context of subjects on suppressive ART with relatively preserved immunity and preexisting HIV-1 specific antibodies [7, 28, 34]. We found, that the multi-clade (A, B, and C) rAd5 vector boost [6], administered at week 32, induced a significant cross clade HIV-1 specific antibody response in vaccinated study participants at week 36, compared to baseline. The majority of the immune response elicited by the vaccine was directed against EnvA and EnvC, whereas antibody signal intensities against EnvB varied only marginally compared to baseline. We believe that the subtle modification in EnvB-specific signals in the vaccine arm is due to the high frequency of preexisting EnvB specific antibody titers in this cohort [28]. In comparison, EnvA and EnvC subtype-specific antibody titers at baseline were almost 4-fold lower than EnvB titers at baseline. Additionally, we noticed a 1.8-fold variation in EnvB specific baseline antibody signal levels between the vaccine and non-vaccine arm even though the groups were randomized.

We detected substantially higher EnvB signal intensities in the non-vaccine arm at week 56, compared to baseline levels, whereas no such trend was observed with EnvA or EnvC specific antibody signals. This may suggest an immune modulating effect of ART intensification with maraviroc and raltegravir. We also speculate that EnvB specific antibody titers in the non-vaccine arm were induced by continuous low-level antigenic stimulation despite maximal anti-viral activity as previously reported by others [35, 36]. Although, signal intensities of the non-vaccine arm did not reach antibody signal levels of the vaccine arm, we found that EnvB intensities were steadily increasing over time, thus reducing the gap between antibody levels.

Nonetheless, it was interesting to find that DNA prime-rAd5 boost immunization did not significantly boost EnvB antibody levels in the vaccine arm. Similarly, the VRC 101 study [20] revealed no significant change in pooled clade A, B, and C envelope antibody titers between serum samples before and after vaccination with the same vaccine product. Although we found significantly elevated cross clade antibody titers, this suggests that the vaccine combination is sub-optimal as a therapeutic option for those already infected with HIV. A recent study revealed that preexisting antigen specific antibody levels can be boosted up to 10-fold by a second booster immunization instead of using only a single dose [37]. This could explain why the EnvB antibody responses were not greater in the vaccine arm of this study.

It is widely accepted that an HIV vaccine should be capable of eliciting potent cross-clade neutralization activity to protect against infection [38–40]. However, the ability to elicit potent and broadly neutralizing antibodies against HIV-1 infection has been a long sought-after but elusive goal of HIV vaccine research so far [41–44]. Previous phase I and phase II clinical trials with DNA prime- and envelope protein boost combinations in healthy individuals revealed only low level neutralizing antibody responses against autologous or Tier 1 viruses [10, 11]. Hence, we expected only low-titer neutralizing antibodies with minor potency against clade A and C viruses after vaccination [45, 46]. Accordingly, we did not assess cross clade neutralization responses of the latter and focused entirely on clade B HIV-1 neutralization efficiency, as we were interested in the evolution of clade B specific neutralization response of our study participants. Based on our data, neutralization potency against the clade B Tier 1 isolate HIV-1_SF162_ improved slightly, however not significantly, after vaccination, whereas IC_50_ values in the non-vaccine arm slowly declined over the study period. It is noteworthy that baseline IC_50_ values in the non-vaccine arm were about 3-fold higher than baseline IC_50_ values in the vaccine arm. We speculate that the difference in baseline IC_50_ levels might derive from lower EnvB specific antibody levels (1.8 fold) in the non-vaccine arm, thus limiting the frequency of neutralizing antibodies in this group. Although antibody levels in the non-vaccine arm constantly increased over time, no positive effect on neutralization was detectable. This could have many reasons including, but not limited to, continuous antigen stimulation by non-native envelope derivatives like gp120 monomers or gp41 monomers or potential epitope masking by non-neutralizing antibodies binding near vulnerable sites [47, 48]. Interestingly, the more neutralization resistant clade B Tier 2 isolate HIV-1_JR-FL_ revealed slightly better IC_50_ levels in the non-vaccine arm. However, the subtle changes in neutralization potency after baseline were similar between either groups, thus indicating no or only minor impact of ART intensification or the vaccine. This was also reflected by the fact that subjects with the highest HIV-1_JR-FL_ IC_50_’s (≥200 μg/mL) at baseline did not improve over time. Ultimately, we believe that most of the marginally enhanced antibody neutralization potency in both study arms is mediated by ongoing somatic mutations and affinity maturation in already existing HIV-1 specific B-lymphocytes [49, 50].

Based on the partial success of the RV144 clinical trial, evaluation of ADCC activity has become an important assessment of HIV-specific antibody function [51–53]. In this study, we found that ADCC activity against HIV-1_SF162_ infected cells followed a similar trend in both study arms. In contrast, BG505 SOSIP.664 gp140 decorated cells showed a slight increase in ADCC activity in the vaccine arm compared to the non-vaccine arm. Since the BG505 SOSIP.664 gp140 envelope trimer derives from a clade A strain it is very likely that the vaccination elicited clade A specific antibodies, which temporarily mediated ADCC activity. In contrast, ADCC activity against HIV-1_JR-FL_ infected cells in the non-vaccine arm was significantly enhanced compared to the vaccine arm. We found this interesting since the elevated antibody levels against EnvB in the non-vaccine arm had minimal impact on the neutralization potency against HIV-1_JR-FL_ but mediated significant antibody effector functions against HIV-1_JR-FL_ infected cells. This indicates that antibody Fc mediated effector functions, beyond neutralization, are important as a protective mechanism [54, 55]. In addition, we found significant inverse correlations between HIV-1 neutralization potency and ADCC activity along with significant positive correlations between antibody signals/titers and ADCC activity. This contrasts with a recently published study [56] where no correlations were found between neutralization, antibody titers, and ADCC.

In summary, we found that DNA prime-rAd5 boost HIV vaccine increased cross clade antibody titers and associations exist between specific antibody signal intensities on microarray chips and neutralization potency. We also showed that ADCC activity was higher in individuals with higher antibody levels. Nevertheless, vaccination did not significantly enhance neutralization potency or ADCC efficacy. These antibody functions are likely required for successful preventive or therapeutic HIV vaccines. A lack of improvement in these functions are additional explanations of why the EraMune 02 strategy failed to reduce total CD4+ cell-associated HIV DNA, as noted in the primary clinical trial [21]. Current therapeutic HIV vaccine strategies may need to be adapted to boost or improve these existing clade B HIV-specific antibody functions. Ultimately, we need additional research on how the immune system evolves during long-term suppressive ART to fully understand the impact of novel immunologic interventions aimed at controlling or eradicating HIV.

## Supporting Information

S1 Fig HIV-1_JR-FL_ neutralization analysis in a subset of subjects with IC_50_ values below 200 μg/mL. We found slightly enhanced neutralization potency against HIV-1_JR-FL_ in both the vaccine arm (n = 6) and the non-vaccine arm (n = 8). However, none of the changes in IC_50_ were significant.
(TIF)

S2 Fig ADCC activity of SOSIP trimer decorated cells in subjects with ADCC levels higher than background. After excluding patients with no ADCC activity against the clade A SOSIP trimer ADCC activity in the non-vaccine arm (n = 9) declined significantly faster at week 56 (p = 0.01) than in the vaccine group (n = 7), compared to baseline activity.
(TIF)

S3 Fig Raw data of chip analysis. Data and sample information has been summarized as a GEO submission file.
(XLSX)
