# Supplementary figures and images for: HIV-1-Specific Antibody Response and Function after DNA Prime and Recombinant Adenovirus 5 Boost HIV Vaccine in HIV-Infected Subjects
Source: PLoS One. 2016 Aug 8;11(8):e0160341. doi: 10.1371/journal.pone.0160341 (PMC4976892; doi:10.1371/journal.pone.0160341)

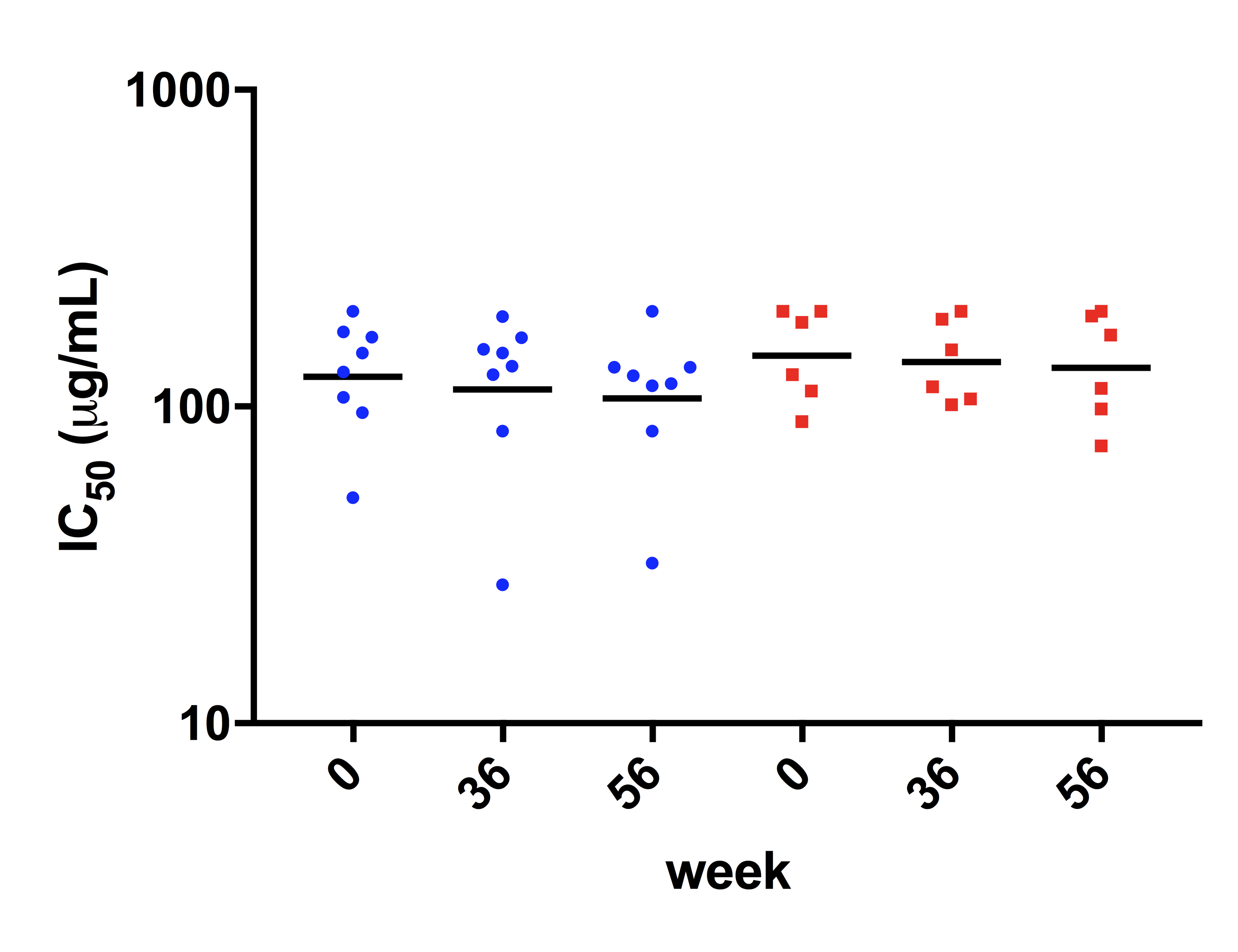

Supplement: S1 Fig — We found slightly enhanced neutralization potency against HIV-1JR-FL in both the vaccine arm (n = 6) and the non-vaccine arm (n = 8). However, none of the changes in IC50 were significant. (TIF) [file pone.0160341.s001.tif]

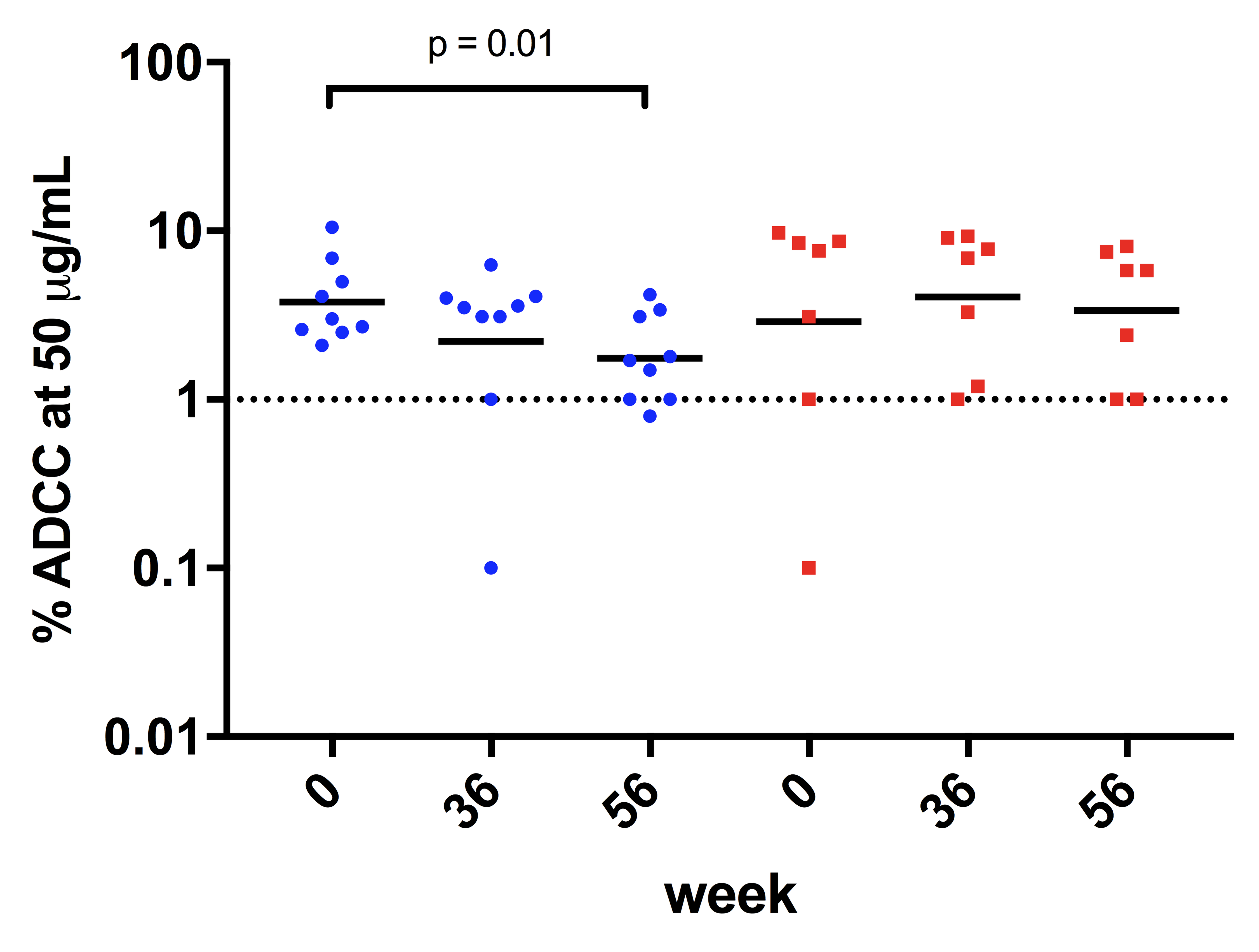

Supplement: S2 Fig — After excluding patients with no ADCC activity against the clade A SOSIP trimer ADCC activity in the non-vaccine arm (n = 9) declined significantly faster at week 56 (p = 0.01) than in the vaccine group (n = 7), compared to baseline activity. (TIF) [file pone.0160341.s002.tif]
